# Community Medical Leadership Workshop: An Introduction to Physician Leadership for Resident Physicians

**DOI:** 10.7759/cureus.20005

**Published:** 2021-11-29

**Authors:** Emmanuel Tito, Sarah Black, Patrick Hilaire, Joseph Weistroffer, Cheryl Dickson

**Affiliations:** 1 Internal Medicine, Western Michigan University Homer Stryker M.D. School of Medicine, Kalamazoo, USA; 2 Orthopaedic Surgery, Western Michigan University Homer Stryker M.D. School of Medicine, Kalamazoo, USA; 3 Medicine, Western Michigan University Homer Stryker M.D. School of Medicine, Kalamazoo, USA; 4 Pediatrics and Adolescent Medicine, Western Michigan University Homer Stryker M.D. School of Medicine, Kalamazoo, USA

**Keywords:** internal medicine residency, physician educators, academic physician, educational leadership, residency leadership, leadership in healthcare management, community development, physician training, academic leadership, medical leadership

## Abstract

Objective

The Community Medical Leadership Workshop (CMLW) aims to prepare residents to become effective physician leaders through medical leadership lectures and case scenario discussions. By the end of the CMLW, participants will be able to define leadership in medicine, employ strategies to manage conflict and differences of opinions in the workplace, demonstrate effective communication skills while working with others, and describe the role of power in effective leadership.

Methods

A total of 32 resident physicians participated in our workshop that is based on the leadership practice inventory (LPI) and the Medical Leadership Competency Framework (MLCF). Our evaluation assessed communication strength, conflict resolution, time management, negotiation, delegation, teamwork, and community service.

Results

Most participants were satisfied with the course. They rated the workshop's contents the highest. In addition, over 90% of learners would recommend this workshop to others. We found a statistically significant increase in learners’ ability to provide opportunities to include patients in quality improvements.

Conclusion

Our workshop was designed and tailored for resident physicians to introduce them to physician leadership. The workshop was well received and could serve as a model to promote qualities in residents that define effective physician leaders.

## Introduction

The term “leadership” has been defined by many and contested, but most authors would agree that a leader inspires, motivates, and implements strategies for individuals and systems in which they work [1]. Throughout the years, the responsibilities of physicians have evolved from caring for patients to becoming leaders and advocates in their local communities [2]. It has become evident that healthcare settings also provide one of the most rewarding opportunities in leadership while making a positive change in patients’ lives. Leadership is an important aspect of a physician's professional career regardless of their field of specialization. While their primary role is to prevent, diagnose, and treat illnesses, physicians have a far-reaching impact on patients’ outcomes and experiences when they are effective leaders. They are ultimately responsible for the overall outcome of the patient in a healthcare team.

Physicians can have a significant impact in improving the quality of service delivered in a healthcare organization. According to a report by the Institute of Medicine, academic health centers should “develop leaders at all levels that can manage the organizational and system changes necessary to improve health through innovation in health professions education, patient care, and research” [2]. It is clear that physicians must develop adequate skills to collaborate with the medical team and effectively lead a team in the patient care community. Leadership skills are crucial to envisioning high-quality patient care, but many physicians have limited exposure to leadership training. In residency programs, there is limited exposure to leadership development for physicians in training. In 2008, the Medical Leadership Competency Framework (MLCF) was jointly developed by the Academy of Medical Royal Colleges and the United Kingdom’s National Health Service (NHS) to direct leadership training for physicians and medical students [3].

As resident physicians spend countless hours in training in their respective specialties, they are often the main medical staff interacting with patients and the rest of the medical team. They truly experience the daily functioning of healthcare services. These medical residents are distinctively placed to understand how patients experience healthcare, and how physicians can improve services for the people. They are uniquely placed to develop experience in leadership through networking with other people, departments, and ways of working. They are also in a position to understand how the patient experiences healthcare, and how the processes and systems of delivering care can be improved [3].

The Association of American Medical Colleges (AAMC) has promoted new roles for physician leaders and a focus on organizational leadership in a new era of health care [4]. However, there is limited exposure in medical residency programs to provide leadership development for resident physicians. While several medical residents seek to be physician leaders, they are unprepared to face the challenges. Barriers to leadership training in residency include time restriction, schedule conflicts, and cost. Though leadership in medicine has been promoted in the past decade, very few works of literature are available on implementing leadership programs that would effectively target medical residents [5,6].

Systematic search and analysis of PubMed and MedEdPORTAL publications revealed that there have been very few successful programs aiming to prepare learners to become physician leaders [7,8]. However, these programs have focused on medical students. One of these publications is a student-led initiative that focused on first-year medical students to prepare them to be physician leaders [7]. Another publication on leadership targeted both residents and students but introduced them to leadership responsibilities and opportunities through the application of the six-step Kern model [8]. Several other MedEdPORTAL publications have focused on developing leadership skills for faculty or a limited number of resident physicians [9,10]. We introduce a leadership training workshop tailored for resident physicians that follows the leadership practice inventory (LPI) and MLCF for the design and implementation [3,10]. This workshop would allow participants to develop individual leadership attributes through medical leadership lectures and case scenario discussions.

By the end of the session, learners will be able to define leadership in medicine, employ strategies to manage conflict in the workplace and differences of opinions, demonstrate effective communication skills while working with others, and describe the role of power in effective leadership.

## Materials and methods

The physician leadership workshop consisted of three parts: a pre-workshop reading assignment to introduce participants to the concept of medical leadership [11,12], medical leadership lectures, and an interactive session to apply newly acquired skills to a variety of case scenarios. Leadership lectures consisted of two separate topics based on workshop objectives: influencing others and defining leadership as well as conflict management. There were six cases that the moderator would present. Cases were real-life challenging cases that require resident physicians to act as physician leaders. The workshop was done virtually to prevent limitations on participation size and to ensure a safe learning experience in the midst of the coronavirus disease 2019 (COVID-19) pandemic. Learners were internal medicine (IM), medicine-pediatrics (Med-Peds), and orthopedic surgery (Ortho) resident physicians.

Evidence-based approaches were implemented to stimulate learners’ engagement and retention. These approaches included individual self-reflection, small and large group discussions, and leadership practice assessments [10]. In preparation for the workshop, moderators were encouraged to spend a minimum of one hour to become familiar with the PowerPoint presentations, facilitator guide (Appendix A), and case scenarios. The facilitator guide identified names of moderators, lecturers, participants’ names, and assigned cases. Moderators were faculty members who would also provide each team feedback during the discussion session. Feedback included comments regarding communication skills, leadership qualities, conflict management within teams, and their personal experience. Lecturers were attending physicians within the residency programs. They were encouraged to review their lecture PowerPoint slides as well as the rest of the materials prior to the workshop.

We designed two separate workshops: the first session for IM and Med-Peds residents and a second reiteration for Ortho residents. Each workshop took approximately 120 minutes, not including the time spent on the pre-workshop reading assignment. The virtual workshop began with a 60-minute lecture presentation followed by a 25-minute small group discussion with participants broken down into smaller groups. IM learners consisted of five groups of four to five participants. Ortho participants consisted of three groups of four to five participants. Small group discussions were designed to allow a dynamic discussion on cases and an optimal workshop outcome. A large group discussion comprising all participants was scheduled for 20 minutes following the small group discussion. This was entailed to have each group select a team leader that would share a key learning experience from the workshop. The last five minutes of the workshop were reserved for closing remarks from moderators and participants.

The workshop was designed to follow the above objectives based on the MLCF outlined by the Academy of Medical Royal Colleges. Survey participants consisted of residents from the Western Michigan University School of Medicine. A Research Electronic Data Capture (REDCap) database was used for data collection and storage. Questionnaires were created using REDCap and were distributed to participants before and after the workshop via email (Appendix B). Data were entered by learners and collected in two web-accessible REDCap surveys. After completing pre-workshop reading assignments introducing medical leadership concepts [11,12], learners completed pre-workshop surveys. Access to the surveys was provided via URL in e-mailed invitations generated by REDCap. Responses were anonymous, and no identifiers or any data identifying the study participants were collected. REDCap's “Participant Identifier” functionality was used to link each subject’s pre and post-survey responses.

We used Kirkpatrick’s model (levels 1 and 2) to evaluate the success of the physician leadership workshop. We employed subjective evidence for levels 1 and 2. To understand level 1, learners completed a specific set of questionnaires to assess their reaction to the workshop. To assess level 2, medical residents completed a pre-workshop and a post-workshop assessment. The pre-workshop assessment was administered to assess baseline medical leadership confidence level prior to the workshop. On this survey, content addressed communication strength, conflict resolution, time management, negotiation, delegation, teamwork, and community service. The post-workshop leadership assessment was the same as the pre-workshop assessment, and it was administered to assess the progression and measure improvement.

Our Institutional Review Board (IRB) determined that the proposed activity (WMed-2021-0791) does not meet the definition of research as defined by the Common Rule and FDA.

## Results

At the two virtual Community Medical Leadership Workshops, there were a total of two lecturers and 10 moderators. They were all faculties of the medical school that have served in various leadership roles throughout their career. For the two leadership conferences, a total of 32 resident physicians and 14 medical students were in attendance. The final dataset contained 32 survey responses with 24 complete surveys. Of 32 resident physician learners, 24 (75%) responded to the pre- and post-workshop leadership assessments. Only those 24 survey results were analyzed. The breakdown of those 24 residents by post-graduate year and specialty is given in Table 1. Because the workshop was tailored for resident physicians, medical students in attendance were not asked to take the survey.

**Table 1:** Breakdown of participants by post-graduate year and specialty.

Post-graduate year	Internal medicine	Medicine-pediatrics	Orthopedic surgery	Total
1	3	1	1	5
2	4	0	3	7
3	3	0	3	6
4	0	0	3	3
5	0	0	3	3
Total	10	1	1	24

**Figure 1:**
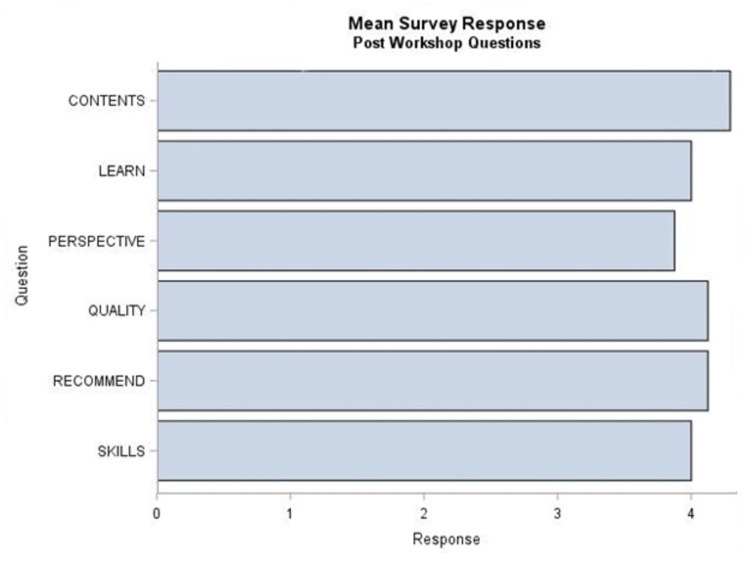
Summary of learner satisfaction with the workshop (Kirkpatrick level 1). Resident physicians rated the extent of agreement or disagreement with each statement as strongly disagree (1), disagree (2), neither agree nor disagree (3), agree (4), and strongly agree (5). CONTENTS - I am satisfied with the contents of the workshop. LEARN - This workshop encouraged me to learn more about the topic. PERSPECTIVE - This workshop changes my perspective on leadership. QUALITY - The quality of this workshop matched my expectations. RECOMMEND - I will recommend this workshop to others. SKILLS - This workshop addressed the skills I need to improve my team.

**Figure 2:**
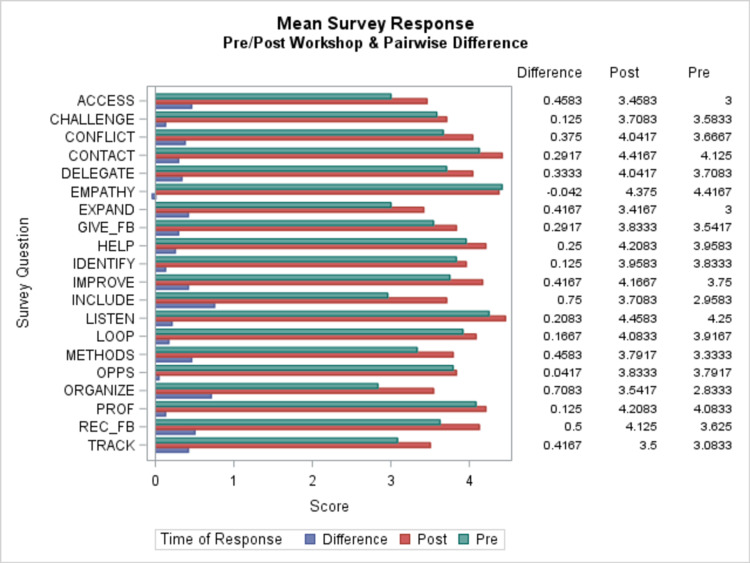
Frequency breakdown and pre-to-post changes for each of the Likert responses as well as a bar graph for each. Resident physicians rated their confidence level in applying leadership skills and knowledge before and after the workshop (Kirkpatrick level 2) in the following scale: not confident at all (1), slightly confident (2), somewhat confident (2), fairly confident (4), and very confident (5). LISTEN - I listen actively to the concerns of my patients. REC_FB - I provide opportunities to receive feedback on my communication skills. OPPS - I provide opportunities for open communication with the community leaders. GIVE_FB - I provide appropriate and honest feedback to fellow health providers. IMPROVE - I contribute actively to further improve the cohesion of my healthcare team. CONFLICT - I provide ideas and methods for conflict resolution when the opportunity arises. DELEGATE - I delegate responsibilities appropriately when the situation allows. PROF - I maintain a high level of professionalism regardless of the situation. LOOP - I maintain closed-loop communication when discussing patient health care. IDENTIFY - I identify opportunities for the education of fellow healthcare providers. INCLUDE - I provide opportunities to include patients in quality improvements. EMPATHY - I demonstrate empathy to my patients regardless of status or background. CHALLENGE - I challenge those that perpetuate injustices in health care. TRACK - I develop methods to identify and track lapses in quality control. ACCESS - I work actively to break down barriers to healthcare access for all my patients. EXPAND - I talk about means of expanding access to health care to those without. ORGANIZE - I organize skill-sharing opportunities between healthcare providers. METHODS - I identify the inefficiencies and communicate methods of improvement. CONTACT - I provide a reliable method for members of my healthcare team to contact me. HELP - I recognize my limitations and seek appropriate help when needed. CONTENTS - I am satisfied with the contents of this workshop. SKILLS - This workshop addressed the skills I need to improve my team. LEARN - This workshop encouraged me to learn more about the topic. PERSPECTIVE - This workshop changed my perspective on leadership. QUALITY - The quality of this workshop matched my expectations. RECOMMEND - I will recommend this workshop to others.

Participants demonstrated high satisfaction with the contents of the workshop and leadership skills learned to improve their team. Over 90% of learners were satisfied with the workshop contents and will recommend this workshop to others. Of the resident physicians, 83% agreed that the quality of the workshop matched their expectations. Figure 1 reveals the overall learner satisfaction in frequency breakdown and bar graphs. The mean for the five-level Likert responses for the 20 questions in both the pre- and post-survey as well as of the pre-to-post difference in each of these questions were obtained in Figure 2. We used a paired sample t-test to determine a statistically significant change in confidence. The Benjamini-Hochberg procedure was used to control the FDR [13].

The initial statistical analysis plan called for keeping the false discovery rate (FDR) at or below 5%. This resulted in only one significant difference: INCLUDE - “I provide opportunities to include patients in quality improvements.” By increasing the allowable FDR to 10%, five more differences become significant: ORGANIZE - “I organize skill-sharing opportunities between healthcare providers”; EXPAND - “I talk about means of expanding access to health care to those without”; ACCESS - “I work actively to break down barriers to healthcare access for all my patients”; REC_FB - “I provide opportunities to receive feedback on my communication skills”; METHODS - “I identify the inefficiencies and communicate methods of improvement.” These results are indicated in Table 2.

**Table 2:** Critical p-values for learner responses to pre- and post-workshop questions. Only statistically significant p-values were shown in the table for FDR at or below 5% and FDR at or below 10%. REC_FB - I provide opportunities to receive feedback on my communication skills. INCLUDE - I provide opportunities to include patients in quality improvements. ACCESS - I work actively to break down barriers to healthcare access for all my patients. EXPAND - I talk about means of expanding access to health care to those without. ORGANIZE - I organize skill-sharing opportunities between healthcare providers. METHODS - I identify the inefficiencies and communicate methods of improvement. FDR - false discovery rate.

Question	p-value	Critical value for FDR = 10%	Critical value for FDR = 5%	Significant difference? (FDR = 10%)	Significant difference? (FDR = 5%)
INCLUDE	0.001139	0.005	0.0025	Yes	Yes
ORGANIZE	0.006527	0.01	0.005	Yes	No
EXPAND	0.009152	0.015	0.0075	Yes	No
ACCESS	0.018257	0.02	0.01	Yes	No
REC_FB	0.019795	0.025	0.0125	Yes	No
METHODS	0.024329	0.03	0.015	Yes	No

## Discussion

Data have shown that patient outcomes are improved with physician leadership [14]. Our results showed that resident physicians increased confidence in their leadership skills. Our workshop was successful because it utilized multiple different educational formats to help learners to retain and implement material learned: large group didactics, small group case discussions, and self-reflection. Resident physicians were engaged, shared their experiences, and related to one another during our case scenario discussions. Another reason for our major success is that the workshop was developed from a pre-existing leadership practice inventory and a medical leadership competency framework that promotes participant-centered learning. We designed a comprehensive leadership assessment tool to clearly measure learning objectives as well as outcomes. We found objective and statistically significant changes in participants’ leadership practices.

Limitations to our study include sample size, single-institution study, and self-reported improvement measures. Time restraints and schedule conflicts in residency are other limitations that may limit the participation of both residents from other medical specialties and volunteer faculties. As for future work, we plan to recruit additional resident physicians across a variety of specialties in our institution. We could potentially assess the extent of behavior change with acquired leadership knowledge and skills (Kirkpatrick level 3). In addition, as a future direction, we will individually track learners’ leadership skills for improvement throughout their time in residency. Another direction is to develop a longitudinal leadership curriculum tailored for residents and expand across other specialties. It has yet to be defined as the optimum method to teach leadership in graduate medical education, so future studies could work to delineate more efficient methods for teaching leadership.

## Conclusions

The Community Medical Leadership Workshop was designed and tailored for resident physicians to introduce them to physician leadership. The medical case scenarios were selected to reflect real-life challenging situations that necessitate residents to act as physician leaders. The workshop was successfully implemented during a two-hour-long grand round without having residents take time off their clinical responsibility. The workshop has been well received with ongoing support from the medical school faculties and residency program directors. CMLW intended to introduce resident physicians to leadership skills to prepare them to be more effective physician leaders. After the workshop, learners were more supportive and related to each other's experiences. We hope that other institutions can replicate our leadership training across multiple medical specialties to help create personal and organizational transformation that benefits patient outcomes.

## Appendices

Appendix A: community medical leadership facilitator guide

This guide contains workshop instructions, agenda, and structure for participants. The guide also identifies the names of moderators, lecturers, participants’ names, and assigned cases.

Overall Outcome

The goal of the workshop is to introduce leadership training to medical resident physicians and to prepare them to become more effective physician leaders.

Educational Objectives

At the completion of this module, learners will be able to:

1. Define leadership in medicine.

2. Employ strategies to manage conflict in the workplace and differences of opinions.

3. Demonstrate effective communication skills while working with others.

4. Describe the role of power in effective leadership.

Timeline

• Pre-workshop reading assignment (60 minutes)

• Introduction of the workshop’s lecturers, materials, and moderators (5 minutes)

• Leadership presentation (60 minutes)

- “Defining leadership and how to influence others” (30 minutes)

- “Conflict management” (30 minutes)

• Break and self-reflection (5 minutes)

- What is your leadership style?

- What actions can I take to be a better physician leader for my community?

• Small group case discussion (25 minutes)

• Large group recap (20 minutes)

- Team leaders share their learning experience

- Moderators’ comments

• Summary and Q&A (5 minutes)

- Closing remarks by lecturers

- Moderators’ final thoughts

Appendix B: community medical leadership evaluation questionnaire

**Table 3:** Pre- and post-workshop assessment questionnaire. Participants were asked to rate their degree of confidence in leadership skills on a five-point Likert scale (1 = not confident at all, 2 = slightly confident, 3 = somewhat confident, 4 = fairly confident, and 5 = very confident). Learners were also asked to rate their reactions to the workshop. This question also used a five-point Likert scale (1 = strongly disagree, 2 = disagree, 3 = neither disagree nor agree, 4 = agree, and 5 = strongly agree). This questionnaire is a modified leadership practice inventory (LPI) tool. Our focus was on the degree of confidence so statements on the LPI were changed to address how well learners thought they could perform particular tasks rather than the frequency with which they completed the task as in the original LPI.

	Strongly disagree	Disagree	Neither agree nor disagree	Agree	Strongly agree
I am satisfied with the contents of this workshop.	1	2	3	4	5
This workshop addressed the skills I need to improve my team.	1	2	3	4	5
This workshop encouraged me to learn more about the topic.	1	2	3	4	5
This workshop changes my perspective on leadership.	1	2	3	4	5
The quality of this workshop matched my expectations.	1	2	3	4	5
I will recommend this workshop to others.	1	2	3	4	5
	Not confident at all	Slightly confident	Somewhat confident	Fairly confident	Very confident
I listen actively to the concerns of my patients.	1	2	3	4	5
I provide opportunities to receive feedback on my communication skills.	1	2	3	4	5
I recognize my limitations and seek appropriate help when needed.	1	2	3	4	5
I provide opportunities for open communication with community leaders.	1	2	3	4	5
I provide appropriate and honest feedback to fellow health providers.	1	2	3	4	5
I contribute actively to further improve the cohesion of my healthcare team.	1	2	3	4	5
I provide ideas and methods for conflict resolution when the opportunity arises.	1	2	3	4	5
I delegate responsibilities appropriately when the situation allows.	1	2	3	4	5
I maintain a high level of professionalism regardless of the situation.	1	2	3	4	5
I maintain closed-loop communication when discussing patient health care.	1	2	3	4	5
	Not confident at all	Slightly confident	Somewhat confident	Fairly confident	Very confident
I Identify opportunities for the education of fellow healthcare providers.	1	2	3	4	5
I provide opportunities to include patients in quality improvements.	1	2	3	4	5
I demonstrate empathy to my patients regardless of status or background.	1	2	3	4	5
I challenge those that perpetuate injustices in healthcare.	1	2	3	4	5
I develop methods to identify and track lapses in quality control.	1	2	3	4	5
I work actively to break down barriers to healthcare access for all my patients.	1	2	3	4	5
I talk about means of expanding access to health care to those without.	1	2	3	4	5
I organize skill-sharing opportunities between healthcare providers.	1	2	3	4	5
I identify the inefficiencies and communicate methods of improvement.	1	2	3	4	5
I provide a reliable method for members of my healthcare team to contact me.	1	2	3	4	5
